# Guidelines for the prevention of travel-associated illness in older adults

**DOI:** 10.1186/s40794-017-0054-0

**Published:** 2017-06-13

**Authors:** Tida K. Lee, Jack N. Hutter, Jennifer Masel, Christie Joya, Timothy J. Whitman

**Affiliations:** 1 Infectious Diseases Service, Walter Reed National Military Medical Center, 8901 Wisconsin Avenue, Bethesda, MD 20889 USA; 2 Naval Medical Research Center, Silver Spring, MD USA

**Keywords:** Travel medicine, Elderly, Pre-travel counseling

## Abstract

International travel to the developing world is becoming more common in elderly patients (defined here as individuals greater than 65 years old). When providing pre-travel counseling, providers must appreciate the changing physiology, comorbidities, immunity and pharmacokinetics associated with the aging process to prepare elderly patients for the stressors of international travel. These guidelines present an evidence-based approach to pre-travel counseling, immunization, and pharmacology concerns unique to elderly patients seeking care in a travel clinic setting.

## Background

The US Department of Commerce estimated that over 73 million Americans took part in international travel in 2015 [1]. Elderly travelers made up 5–10% of US international travelers over the last few years [1–3] and will likely be a growing number as the US Census Bureau projects near doubling of the elderly population over the next 40 years [4]. With this growing elderly population, the number of retired or entering retirement will likely continue to result in a rise in the numbers of elderly travelers who would benefit from pre-travel counseling [5, 6].

The aging process brings with it physiological changes and an increased incidence of underlying medical conditions [7] which may put older travelers at higher risk for common travel-associated disease processes [8]. For example, the natural changes in body composition, hearing and vision seen with aging can lead to frailty making older travelers more susceptible to falls, especially as travelers are in new environments and may not be familiar with their surroundings [7, 9]. Reduced functional reserve and homeostatic dysregulation seen in the aging process could lead to increased risk for altitude illness, heat injury and dehydration during travel [8–11]. Furthermore, with the aging immune system, there is evidence of waning immunity from some vaccines received in the past, and diminished responses to more recent immunizations, which may place the elderly population at increased risk to otherwise vaccine-preventable infectious diseases [12].

There have been several publications with recommendations for elderly travelers in the past, but as they are outdated [6, 8, 9], these guidelines serve to take an updated review of the literature and make evidence based recommendations specific to the elderly traveler (age ≥ 65), providing strength and rationale for these recommendations.

These guidelines are broken down into three sections that focus on key aspects of travel medicine for elderly patients; pre-travel counseling for general travel topics, immunization, and unique medication concerns (malaria prophylaxis, travelers’ diarrhea treatment, etc.) and present these recommendations followed by a summary of the evidence. To develop the guidelines, we performed a comprehensive literature search on travel medicine for the elderly across multiple databases. We searched the PubMed for relevant articles, using the following terms: “elderly” AND “travel medicine”, “immunizations”, “vaccinations”, “cardiovascular disease”, “pulmonary disease”, “malignancy and thromboembolic”, “pneumococcal vaccine”, “Tdap”, “zoster vaccine”, “influenza vaccine”, “yellow fever”, “hepatitis A”, hepatitis B”, “rabies”, “typhoid”, meningococcal”, “polio”, “Japanese encephalitis”, “travelers’ diarrhea”, “jet lag”, “altitude sickness”, “malaria”, “travelers’ diarrhea”, “jet lag”, and “altitude illness”.

We used the GRADE system to grade both the strength of the recommendations and the quality of the evidence [13]. The strength of the recommendation is graded as “strong”, when the evidence shows the benefit of the intervention or treatment clearly outweighs any risk, and as “conditional”, when uncertainty exists about the risk-benefit ratio. The quality of the evidence is graded as follows: “high”, if further research is unlikely to change our confidence in the estimate of the effect; “moderate”, if further research is likely to have important impact and may change the estimate; and “low”, if further research is very likely to change the estimate;” very low”, if an effect is very uncertain (Table 1).

**Table 1:** Summary and strength of recommendations

Pre-travel counseling
1. Elderly patients who anticipate overseas travel should meet with a provider familiar with travel medicine to undergo risk assessment and guidance. (Strong recommendation, moderate-quality evidence)
Assessment of Co-morbidities
2. Cardiovascular Disease: Elderly patients with a history of coronary artery disease (CAD) should be evaluated for acute or recent cardiac diagnoses prior to travel. (Strong recommendation, moderate-quality evidence)
3. Pulmonary Disease: Elderly patients with underlying chronic pulmonary disease such as COPD and emphysema, or acute pulmonary disease such as pneumonia should discuss travel plans with a clinician about risks of exacerbation and should contact airlines in advance if there is a need for supplemental oxygen during the flight. (Strong recommendation, moderate quality evidence)
4. Malignancy & Thromboembolic: Elderly patients at increased risk for venous thromboembolism (VTD) should consider the use of well-fitted below-the-knee compression hosiery or subcutaneous enoxaparin before and one day after when undertaking journeys of greater than three hours. (Strong recommendation, moderate-quality evidence)
Vaccines
5. General Vaccines: Clinicians should be cognizant that as the immune system ages, it undergoes characteristic changes referred to as immunosenescence which leads to a decline in the protective efficacy from vaccinations and a shortened duration of protection. Strong recommendation, high quality evidence)
6. Non-travel related immunizations: Ensure elderly travelers are up to date on non-travel related immunizations to include pneumococcal pneumonia (pneumovax® and prevnar 13®), tetanus, diphtheria with acellular pertussis (Tdap), live attenuated herpes zoster vaccine. (zostavax®) and seasonal influenza. (Strong recommendation, high-quality evidence)
7. Yellow Fever: The yellow fever vaccine should only be considered for elderly travelers to endemic regions and recognize those who require proof of immunization for travel. Clinicians should weigh the risks and benefits in the context of the individual traveler prior to vaccination. (Strong recommendation, high quality evidence)
8. Hepatitis A: Two doses of hepatitis A vaccine should be given to elderly travelers. (Strong recommendation, high quality evidence)
9. Hepatitis B: The Hepatitis B vaccine should be given to elderly travelers who are at risk for acquiring the disease (e.g. utilizing health care, at risk for blood borne exposure). (Strong recommendation, high quality evidence)
10. Rabies: Individual risk assessment should be made concerning rabies pre-exposure prophylaxis for the traveler. Such risk assessment should include factors such as outdoor exposure risk, rabies endemicity of the region, and access to medical care in country. (Strong recommendation, high quality evidence)
11. Typhoid: Purified Vi Polysaccharide Parenteral vaccine or Ty21a Live-Attenuated Oral vaccine should be considered for travelers to endemic regions. (Strong recommendation, moderate quality evidence)
12. Meningococcal: The conjugated meningococcal vaccine should be considered, and may be a requirement, for elderly travelers to going to regions endemic (e.g. Hajj) with *N. meningitides.* Endemic regions (e.g. Hajj). (Strong recommendation, very low quality evidence)
13. Polio: The polio vaccine should be considered and documentation of vaccination may be required for travelers to endemic regions. (Strong recommendation, moderate quality evidence)
14. Japanese Encephalitis: The Japanese encephalitis vaccine should be given to elderly patients traveling to endemic regions. (Strong recommendation, moderate quality evidence)
Travel Specific Concerns
15. Travelers’ Diarrhea: Treatment of travelers’ diarrhea in elderly patients should be reserved for severe cases. (Strong recommendation, high quality evidence)
16. Jet Lag: In adult patients traveling eastbound on journeys greater than five time zones, melatonin taken for 2 days prior to departure and for 3 days after arrival at the bedtime of the target destination can shorten the duration of jet lag, and in the elderly is safer than using hypnotics or benzodiazepines.; Conditional recommendation, low quality of evidence)
17. Altitude: When traveling to regions at elevation greater than 8200 ft (2500 m), elderly patient should be educated about the effects of altitude illness and prescribed acetazolamide for disease prevention but avoided in patients on high dose aspirin (325 mg daily). (Strong recommendation, moderate quality evidence)
18. Travel Insurance: Elderly patients should be counseled to review their medical insurance policies to see overseas coverage and consider purchasing travel insurance prior to travel as many domestic insurance policies will not cover international aeromedical evacuation. (Conditional recommendation, low quality evidence)
Malaria
19. Malaria Prevention: Ensure elderly travelers are well educated about the importance of adhering to personal protective measures and to present early for care if they develop fevers following travel to malaria endemic regions. (Strong recommendation, high quality evidence)

## Pre-travel counseling; general health

### *Recommendation* - PRE-TRAVEL COUNSELING

1. Elderly patients who anticipate overseas travel should meet with a provider familiar with travel medicine to undergo risk assessment and guidance. (Strong recommendation, moderate-quality evidence).

#### Summary of the evidence

With age, there are several issues that should be considered; preexisting health conditions, waning immunity, vaccine responsiveness and risks, the potential for an alteration in cognitive function, and drug-drug interactions with medications prescribed for many common chronic conditions [8, 11, 14, 15].

## Assessment of co-morbidities

### *Recommendation* – CARDIOVASCULAR DISEASE

2. Elderly patients with a history of coronary artery disease (CAD) should be evaluated for acute or recent cardiac diagnoses prior to travel (Strong recommendation, moderate-quality evidence).

#### Summary of the evidence

Cardiovascular events on an air plane are a common cause of medical incidents in flight and flight diversion with exacerbations in CAD being one of the leading occurances [16–18]. With these cardiovascular disorders clinicians must consider the physiological pressures associated with air travel such as hypobaric hypoxemia and how this may exacerbate underlying cardiovascular disease [19]. This is recognized by the International Air Transportation Association who has published restrictions on various cardiovascular disorders [20].

### *Recommendation* – PULMONARY DISEASE

3. Elderly patients with underlying chronic pulmonary disease such as COPD and emphysema, or acute pulmonary disease such as pneumonia should discuss travel plans with a clinician about risks of exacerbation and should contact airlines if there is need for supplemental oxygen during the flight (Strong recommendation, moderate quality evidence)

#### Summary of the evidence

With air travel, the rise in altitude results in a drop in the PO2 resulting in a hypobaric hypoxemia, which is not a problem for healthy individuals to adapt to, but in elderly individuals with lung disease, this may exacerbate their underlying disease [19, 21]. For individuals that may need supplemental oxygen during the flight, they should work with their healthcare provider and contact the airline to coordinate as travelers are not allowed to carry personal oxygen tanks aboard commercial aircraft per Federal Aviation Administration policy.

### *Recommendation* – MALIGNANCY & THROMBOEMBOLIC

4. Elderly patients at increased risk for venous thromboembolism (VTD) should consider the use of well-fitted below-the-knee compression hosiery or subcutaneous enoxaparin before and 1 day after when undertaking journeys of greater than three hours. (Strong recommendation, moderate-quality evidence).

### Summary of the evidence

Blood flow stasis associated with long duration travel is a weak risk factor for the development of VTD. However, the risk of travel-related thrombosis is higher in individuals with pre-existing risk factors such as older age, and conditions often found in the older population to include malignancy, recent surgery and history of prior blood clots [22]. Travelers at the highest risk of travel-related thrombosis undertaking journeys of >3 h may benefit from well fitted below knee compression stockings and subcutaneous enoxaparin before and 1 day after their flight [20, 22–24].

## Vaccines

### *Recommendation –* GENERAL VACCINE RECOMMENDATIONS

5. Clinicians should be cognizant that as the immune system ages, it undergoes changes referred to as immunosenescence, which lead to a decline in the protective efficacy from vaccinations and a shortened duration of protection. (strong recommendation, high quality evidence)

#### Summary of the evidence

As the immune system ages, the most notable event is the loss of the thymic cortex and medulla to the extent that by the age of 50 years, around 80% of the gland is gone [25]. As a result, the output of mature T cells from the thymus decreases with age which leads to reduced response rates to vaccines.

In addition, B cells also undergo age related changes that impair their immune response and the end result of the decline both B and T cell numbers and function is a reduction in the quantity and diversity of antibody responses to both infections and vaccinations [26, 27].

Despite what is known about vaccines having decreased immunogenicity in elderly patients, there are no alternative schedules for most vaccines to compensate for immunosenescence.

### *Recommendation –* NON-TRAVEL RELATED IMMUNIZATIONS

6. Ensure elderly travelers are up to date on non-travel related immunizations to include pneumococcal pneumonia (pneumovax® and prevnar 13®), tetanus, diphtheria with acellular pertussis (Tdap), live attenuated herpes zoster vaccine (zostavax®) and seasonal influenza. (Strong recommendation, high-quality evidence)

#### Summary of the evidence

The travel clinic visit is an opportunity to review and administer routine immunizations. Five vaccines are currently recommended by the Advisory Committee for Immunizations Practices (ACIP) for persons age 65 and older: seasonal influenza, Tdap, Prevnar 13®, Pneumovax® and Zostavax® [28].

Elderly patients in the US have relatively low rates of vaccine coverage, with only 62.3% receiving pneumonia vaccine, 54.4% tetanus (with or without acellular pertussis), and 15.8% of adults 60 or older with shingles vaccination as assessed via the National Health Interview Survey (NHIS) in 2011. The pre-travel visit is an ideal opportunity to address what may have been overlooked in the primary care setting [29].

As influenza is the most common vaccine preventable infection in travelers, it’s critical that this vaccine is not over looked during the travel visit for elderly patients [30, 31]. A recent meta-analysis demonstrated efficacy in preventing laboratory confirmed influenza in elderly persons during regional and widespread outbreaks, regardless of vaccine match or mismatch to circulating viruses [32].

Addressing the concern immunoscenescence of the influenza vaccine, Langley et al. conducted a randomized controlled trial with a H5N1 A/Indonesia/05/2005 vaccine showing that seroprotection rates in subjects >65 years old 42 days post vaccination was 74% versus 91% in younger adults. Of note, subjects greater than 75 years old had similar seroprotection rates as those aged 65–75 years old [33].

In addition, in two observational studies the immunogenicy of Fluzone®, Fluzone High-Dose® and Fluzone Intradermal® was evaluated in adults less than 60 years old and adults between 61 and 86 year of age. The percentage of subjects with post-vaccination seroconversion for the H1N1 strain was 54% versus 23%, H3N2 strain 79% versus 68%, and B strain 38% versus 11%. [34].

Considering the high risks of acquiring influenza during travel due to crowded airplanes, limited opportunities for hand washing etc. and the low costs and favorable side effect profile, it’s essential that this vaccine be administered to elderly patients.

However, as the influenza vaccine efficacy is somewhat limited in older adults, it is not unreasonable to offer elderly travelers a self-treatment course of oseltamivir or zanamivir if traveling to areas where influenza activity is occurring [35].

Although little is known about the rates of pneumococcal pneumonia in travelers, community-acquired pneumonia (CAP) results in almost 400,000 hospitalizations in the US annually with a case fatality rate of 5–7% [36]. *Streptococcus pneumoniae *is the leading cause of CAP with the burden of the disease clearly being seen in the elderly [36].

The ACIP released new recommendations for pneumococcal vaccination in elderly persons in 2014 advising both pneumovax® and prevnar 13® be given to all adults ≥ age 65 [37]. Considering that rates of compliance with this recommendation has been 62% in the outpatient setting, the pre-travel counseling visit is an excellent opportunity to assure these immunizations have been addressed [29].

Although little is known about rates of herpes zoster outbreaks for travelers, the CDC estimates that approximately 500,000 elderly patients in the US develop this illness every year making it possible for an outbreak to occur during travel [38] Beginning in 2008, the ACIP has recommended a single dose of Zostavax® for all adults older than 60 years however as of 2014, only 28% of adults reported being vaccinated [29]. Of note, more recent data is suggesting that that vaccine efficacy for incidence of herpes zoster wanes significantly after 8 years. [39]. In light of these findings, giving a traveler a booster dose of Zostavax® would be reasonable to consider if an elderly patient’s last dose was greater than 8 years ago.

Little is known about the incidence of tetanus, pertussis and diphtheria in travelers although there has been a considerable increase in rates of pertussis in the US for multiple reasons (increase testing, better diagnostics and shifting genomics in emerging strains) [40].

Kaml et al. demonstrated that booster vaccinations against tetanus, pertussis and diphtheria resulted in a less robust response in subjects over 65 years of age compared to younger individuals. In addition, the immune response to all three components of the vaccine was better in those elderly patients who had been previously been vaccinated against Tdap which reinforces the concept of regular booster vaccinations throughout life [26, 41].

Finally, there has been a focus on vaccinating travelers against measles, mumps and rubella (MMR) vaccination in light of several highly publicized outbreaks in the US linked to non-immune travelers arriving in the country from the developing world [42]. As individuals born prior to 1957 are considered to have acquired immunity against MMR due to natural infection, and this birth cohort essentially encompasses all adults currently 60 or older, the MMR vaccine is usually not a concern during the pre-travel visit for these three infections [43].

### *Recommendation –* YELLOW FEVER

7. The yellow fever vaccine should only be considered for elderly travelers to endemic regions and those who require proof of immunization for travel. Clinicians should weigh the risks and benefits in the context of the individual traveler prior to vaccination. (Strong recommendation, high-quality evidence)

#### Summary of the evidence

Incidence of yellow fever illness in unvaccinated travelers to Africa is estimated at 1 per 2000 for a typical 2 week trip with a risk of death estimated at 1 in 10,000, and the risk is estimated to be 10 times lower in South America. [44].

The live-attenuated yellow fever vaccine is a highly effective vaccine with nearly all recipients (97–100%) developing a neutralizing antibody response [45]. Side effects are rare, but can be severe, and elderly recipients are at increased risk [45–48]. The two feared adverse reactions are yellow fever vaccine-associated viscerotropic disease (YEL-AVD) and yellow fever vaccine-associated neurologic disease (YEL-AND) [48]. The viscerotropic disease results from 17D viremia and is syndromically similar to yellow fever, while the yellow fever vaccine-associated neurologic disease can have various manifestations, such as meningoencephalitis, acute disseminated encephalomyelitis (ADEM), or Guillan-Barré syndrome (GBS) [46]. These events have been described only with primary vaccination [44]. Naturally occurring yellow fever has a higher likelihood of causing severe and fatal infection in the elderly, but more travelers were noted to have died from YEL-AVD than yellow fever itself in a 2012 review [47]. For this reason, a number of factors should be weighed before recommending vaccination to elderly travelers including transmission intensity of yellow fever at the destination, duration of travel, likelihood of mosquito exposure, and plans for future travel to endemic regions. A medical waiver should be issued when risk is judged to outweigh benefit of vaccination.

In February 2015, the ACIP approved a new recommendation that a single dose of yellow fever vaccine provides long-lasting protection and is adequate for most travelers thereby eliminating the need for booster doses in elderly patients and the potential risk of adverse reactions and additional cost [49].

### *Recommendation –* HEPATITIS A

8. Two doses of hepatitis A vaccine should be given to elderly travelers. (Strong recommendation, high quality evidence)

#### Summary of the evidence

Hepatitis A virus (HAV) is a worldwide health concern and behind influenza, is the second most common vaccine-preventable travel-associated infectious disease with an estimated incidence rate of 30 per 100,000 people per month [50]. HAV causes mild illness in children but exposure in unvaccinated adults can cause severe illness, with higher morbidity and mortality in elderly adults [51].

Since its introduction in the mid-1990s, the hepatitis A vaccine has produced a well-documented, robust immune response in children and adults [51]. More recently, D’Acremont et al. compared the immune response of subjects 18–45 years old to a cohort over 50 years old and showed the seroprotection rates in the younger and older subjects were 100 and 65% after the first vaccination and 100 and 97% after the booster [52]. This data shows that in light of immunosenescence, as long as elderly patients receive two doses, hepatitis A is a highly efficacious vaccine in this patient population.

### *Recommendation –* HEPATITIS B

9. The Hepatitis B vaccine should be given to elderly travelers who are at risk for acquiring the disease (e.g. utilizing health care, at risk for blood borne exposure). (Strong recommendation, high quality evidence)

#### Summary of the evidence

The incidence rate of newly acquired hepatitis B infection per 100,000 travelers is estimated to be 25–425 cases per month [53] with nearly 240 million people worldwide chronically infected [54]. In the US, the hepatitis B vaccine for elderly is available by itself or in combination with hepatitis A.

Van Der Meeren et al. conducted a pooled analysis of clinical trial data from healthy adults age > 20 years in 11 studies since 1996 and found an observed protection rate (defined as an anti-HBV surface antigen antibody concentration ≥ 10 mIU/mL) was 98.6% in subject age 20–24 years but only 64.8% in those at age ≥ 65 years [55]. So, although we see significant impact of immunosenescence with the hepatitis B vaccine, it’s reasonable to offer this vaccine to long-term travelers at risk to blood borne exposure with the caveat that there is a known degree of vaccine failure in older travelers.

### *Recommendation -* RABIES

10. Individual risk assessment should be made concerning rabies pre-exposure prophylaxis for the traveler. Such risk assessment should include factors such as outdoor exposure risk, rabies endemicity of the region, and access to medical care in country. (Strong recommendation, high-quality evidence)

#### Summary of the evidence

Over the last decade, 22 confirmed rabies cases have been reported in travelers, and monthly incidence of potential rabid bites vary regionally from 0.2 per 1000 tourists to 23.1 per 1000 tourists. [56]. The WHO estimates that up to 99% of the human rabies cases are transmitted by the bite of an infected dog [56, 57]. Endemicity maps are available through the WHO website to access for risk counseling prior to travel [58].

Concerning rabies vaccines, RabAvert® and Imovax Rabies ®, there is a good body of literature looking at seroconversion rates in patient up to age 65 showing greater than 94% efficacy [59, 60]. In addition, a study investigating the duration of neutralizing antibodies following pre- or post-exposure rabies vaccine in a cohort of patients ranging up to age 78 showed long lasting immune response in the majority of patients [61].

### *Recommendation -* TYPHOID

11. Purified Vi Polysaccharide Parenteral vaccine or Ty21a Live-Attenuated Oral vaccine should be considered for travelers to endemic regions. (Strong recommendation, moderate quality evidence)

#### Summary of the evidence

The incidence rate of typhoid in high risk travelers is estimated to be 3–30 per 100,000 people per month with notably highest incidence in travelers to the Indian Subcontinent [62]. Although the vaccine was not studied specifically in elderly patients, the effectiveness in overseas travelers has been reported to be 65% to 80% in studies from the UK and US, respectively [63, 64]. As such, vaccination of travelers to endemic areas would offer at least moderate protection from the disease.

### *Recommendation -* MENINGOCOCCAL

12. The conjugated meningococcal vaccine should be considered, and may be a requirement, for elderly travelers going to regions endemic (e.g. Hajj) with *N. meningitidis*. (Strong recommendation, very low-quality evidence)

#### Summary of the evidence

It is estimated that the incidence rate in travelers varies widely 0.05–50 per 100,000 people depending on destination with notable high rates in certain geographic regions such as the African Meningitis belt [53]. In the endemic season in these regions, mortality rates have declined with improved vaccination efforts, but suspected meningitis deaths remain greater than 1000 deaths in both the 2013 and 2014 seasons [65].

Preventive vaccination is recommended, and for some regions required, for travelers to these high risk regions, specifically to Hajj [66]. In the US, three meningococcal vaccines are currently available and all are polysaccharide-protein conjugated (Menactra®, Menveo®, and MenHibrix). The polysaccharide vaccine was the only licensed meningococcal vaccine for adults aged ≥56 years but is no longer available in the US.

As such, the CDC recommends the conjugated vaccine for people aged ≥56 who plan on going to regions endemic with *N. meningitides*, but with the recognition that it is not licensed for this age group [67].

### *Recommendation -* POLIO

13. The polio vaccine should be considered, and documentation of vaccination may be required for travelers to endemic regions. (Strong recommendation, moderate quality evidence)

#### Summary of the evidence

Despite eradication of polio in the Americas and progress in global eradication, significant outbreaks have occurred in 2012 to 2014 in seven African and Middle Eastern countries, and more recently two new cases in July 2016 in Nigeria [68].With these ongoing outbreaks, the WHO continues to update polio vaccine recommendations for international travelers to and from certain regions. [69].

Evaluation of booster vaccination with a multivalent vaccine containing tetanus, dipththeria, pertussis, and polio antigens in healthy elderly persons (age > 60 years) compared to younger control group demonstrated that pre- and post-booster antibody concentrations against polio were above protective levels for all three polio strains in most of those elderly individuals analyzed [41]. As such, while polio outbreaks remain a risk in certain regions, providers should assure that elderly patients are up to date with these requirements.

### *Recommendation –* JAPANESE ENCEPHALITIS

14. The Japanese encephalitis vaccine should be given to elderly patients traveling to endemic regions. (Strong recommendation, moderate quality evidence)

#### Summary of the evidence

Japanese encephalitis is the most important cause of viral encephalitis in Eastern and Southeast Asia, affecting about 25 Asian countries and occurring primarily in children but also seen in the elderly as natural immunity wanes over time [70–72]. In 2009, a cell culture-derived killed-inactivated vaccine (Ixario) was approved for the prevention of Japanese encephalitis (JE) in adults >17 years old and had considerably less side effects than the mouse brain-derived killed-inactivated (JE-VAX) making this vaccine easier to give to patients of all ages in the pre-travel setting [72].

Cramer et al. recently completed an open-label, multicenter study showing that Ixiaro was well tolerated in a cohort of patients age 64–83 years and conferred a seropretection rate of 65% 42 days after the second dose [73].

## Travel specific concerns

### *Recommendation* – TRAVELERS DIARRHEA

15. Treatment of travelers’ diarrhea in elderly patients should be reserved for severe cases. (Strong recommendation, high-quality evidence)

#### Summary of the evidence

Recent guidelines have been published for the treatment of travelers’ diarrhea by Riddle et al. recommending the use of antibiotics in cases of severe travelers’ diarrhea with the new definition of severe travelers’ diarrhea as acute diarrhea “that is incapacitating or completely prevents planned activities and all dysentery.” [74] While these guidelines are not age specific, they are certainly applicable to the elderly population as there are several outcomes of travelers’ diarrhea that should be considered in this population. First, if travelers lack an available empiric treatment, they may seek local treatment options, which depending on location there may be a risk of substandard or falsified medications [75] as well as polypharmacy and high rate of invasive procedures [76]. As such, this can result in delay of treatment, possible increased risks of nosocomial infections, and risks for drug-drug interactions when prescribing in elderly with multiple comorbidities on multiple chronic medications (see drug-drug interactions, Table 2) [75, 76]. Second, increased risk of extended spectrum beta-lactamase (ESBL) carriage has been observed after international travel, particularly with higher risk seen with travel to Asia and Africa [77]. Antibiotic use has also been identified in some studies as a risk factor for colonization [77, 78]. This higher risk for ESBL carriage is concerning in the elderly as it may cause further complications for further infections that elderly are often at higher risk for such as urinary tract infections and prostatitis [79, 80]. Third, while the elderly traveler is generally at decreased risk of post infectious irritable bowel syndrome (PI-IBS) than younger travelers, it is still evident that travelers with severe disease are at higher risk of PI-IBS [81]. As such, providing an empiric treatment for severe cases in the elderly may lessen the risk of chronic health consequences such as PI-IBS in this population. Furthermore, antibiotic options must be carefully considered with consideration of FDA fluoroquinolone antibiotic use warnings [82]. In light of this data, antibiotics for severe cases of travelers’ diarrhea is recommended, and consideration for single dose a zithromycin therapy to shorten antibiotic pressure duration [83].

**Table 2:** Drug-Drug Interactions: This table is not completely inclusive, but rather meant to highlight drug-drug interactions between antimalarial drugs, travelers’ diarrhea antibiotics, altitude illness prevention medications and other common drugs prescribed in the elderly

DRUG	DRUGS INTERACTING	RECOMMENDATION	ADVERSE EFFECTS
ANTIMALARIALS			
Mefloquine	Carbamazepine, Phenytoin, Phenobarbitol, Valproic acid	Consider therapy modification	Diminished effect of antiepileptic. Mefloquine contraindicated for malaria prophylaxis in patients with history of seizure disorder.
	Citalopram, Fluoxetine	Consider therapy modification	QTc prolongation
	Azithromycin	Monitor therapy	QTc prolongation
	Diltiazem, Verapamil, Carvedilol	Monitor therapy	Increased serum concentrations of mefloquine
	Compazine	Monitor therapy	Increased serum concentrations of Compazine
	Warfarin	Monitor therapy	May increase anticoagulant effect
Chloroquine	Amiodarone, Fluoxetine, Sotalol	Avoid combination	High risk QTc prolongation
	Cyclosporine	Consider therapy modification	May increase serum concentrations of cyclosporine
	Paroxetine, Ritonavir, Lopinavir, Ketaconazole, Fluconazole	Consider therapy modification	May increase serum concentration of chloroquine
	Ciprofloxacin, Levofloxacin, Azithromycin	Consider therapy modification	QTc prolongation
	Carvedilol, Propranolol, Metoprolol	Monitor therapy	May increase serum concentrations of beta-blocker
	Digoxin	Monitor therapy	May increase serum concentrations of digoxin
	Tacrolimus	Monitor therapy	QTc prolongation
Atovaquone-Proguanil	Ritonavir, Rifampin, Riabutin, Rifapentine	Avoid combination	May decrease the serum concentration of Atovaquone
	Efavirenz, Metoclopramide	Consider therapy modification	May decrease the serum concentration of Atovaquone
	Compazine	Monitor therapy	May increase serum concentration of Compazine
	Warfarin	Monitor therapy	May increase anticoagulant effect
Doxycycline	Calcium salts, Carbamazepine, Phenytoin	Consider therapy Modification	May decrease serum concentrations of doxycycline
	Methotrexate	Monitor therapy	May increase serum concentrations of methotrexate
	Warfarin	Monitor therapy	May increase anticoagulation effect
TRAVELERS’ DIARRHEA ANTIBIOTICS			
Azithromycin Fluoro-quinolones	Citalopram, Fluoxetine, Escitalopram, Amiodarone, Dronedarone	Avoid combination	High risk QTc prolongation
	Atorvastatin, simvastatin	Monitor therapy	May enhance myopathic (rhabdomyolysis) effect
	Warfarin	Monitor therapy	May increase anticoagulant effect
	Multivitamins/Minerals (ADEK, folate, iron), Calcium salts	Consider therapy modification^a^	May decrease serum concentrations of quinolone
ALTITUDE SICKNESS PREVENTION			
Acetazolamide	Brinzolamide, dorzolamide	Avoid combination	Increased risk for metabolic acidosis and nephrolithiasis
	Topirimate, zonisamide	Avoid combination	Increased risk for metabolic acidosis and nephrolithiasis
	Aspirin (>81 mg/day), bismuth subsalicylate	Consider therapy Modification	Metabolic acidosis
	Tramadol, oxycodone, hydromorphone, codeine	Monitor therapy	Risk of orthostatic hypotension
	Dextroamphetamine/amphetamine	Monitor therapy	Decreased excretion of amphetamines
	Lithium	Monitor therapy	Increased lithium excretion
	Metformin	Monitor therapy	Increased risk of lactic acidosis
	Quinidine	Monitor therapy	Decreased excretion of quinidine

^a^Interaction can be minimized by timing of dosing

### *Recommendations* – JET LAG

16. In adult patients traveling eastbound on journeys greater than five time zones, melatonin taken for 2 days prior to departure and for 3 days after arrival at the bedtime of the target destination can shorten the duration of jet lag, and in the elderly is safer than using hypnotics or benzodiazepines. (Conditional recommendation, low quality evidence)

#### Summary of the evidence

People older than 60 have greater difficulty recovering from jet lag, particularly on eastbound flights, due to decreased and irregular melatonin rhythms [84]. A Cochrane review of 10 trials showed efficacy in 8 of the 10 for doses of 0.5 mg–5 mg of melatonin taken at the target bedtime of the destination, with shorter sleep latency for the 5 mg dose, but did not look at the elderly specifically [85]. Zolpidem use in the preceding 180 days was associated with hip fracture with an adjusted odds ratio of 1.95, which was greater than the adjusted odds ratio for benzodiazepines of 1.46 in a large case control study of hip fractures in patients over 65 [86].

### *Recommendation* –ALTITUDE

17. When traveling to regions at elevation greater than 8200 ft (2500 m), elderly patient should be educated about the effects of altitude illness and prescribed acetazolamide for disease prevention but avoided in patients on high dose aspirin (325 mg daily). (Strong recommendation, moderate-quality evidence)

#### Summary of the evidence

In a study comparing elderly (>60) versus younger (20–30 year olds) travelers, elderly travelers do not appear to be limiting their activities despite having significantly more underlying medical conditions, as elderly travelers are noted to be participating in mountain travel and utilizing acetazolamide therapy [87]. Gautret et al. suggested that advanced age may be a risk factor for the development of high-altitude illness when evaluating the GeoSentinel clinic data base of ill travelers presenting for care [21]. However, there are limitations to this numerator-only data base. From this data, we propose that travel medicine providers be aware of a possible connection between advanced age and high-altitude illness and counsel patients about preventative measures and the use acetazolamide for prophylaxis with one exception. There have been numerous case reports of acetazolamide toxicity in patients on high dose aspirin [88]. Pharmacokinetic studies demonstrate that salicylate appears to competitively inhibit plasma binding of acetazolamide, decreasing renal tubular secretion of the drug so if aspirin is needed for heart disease prevention, it should be dosed as 81 mg daily.

### *Recommendation* – TRAVEL INSURANCE

18. Elderly patients should be counseled to review their medical insurance policies for overseas coverage and consider purchasing travel insurance prior to travel as many domestic insurance policies will not cover international aeromedical evacuation. (Conditional recommendation, low-quality evidence)

#### Summary of the evidence

Use of international aeromedical evacuation is expanding in recent years due to an increasing number of travelers, many of whom are elderly or who have preexisting illness, and who are visiting regions of the world with limited availability of local medical resources [89]. There can be significant costs associated with such medical assistance and delays in medical care can be delayed due to lack of guarantee of payment for these services.

## Malaria

### *Recommendation* – MALARIA PREVENTION

19. Ensure elderly travelers are well educated about the importance of adhering to personal protective measures and to present early for care if they develop fevers following travel to malaria endemic regions. (Strong recommendation, high-quality evidence)

#### Summary of the evidence

The Centers for Disease Control and Prevention identify three traditional patient populations as having increased morbidity and mortality from malaria; young children, pregnant women, and travelers coming from areas without malaria visiting malaria endemic regions [90]. Checkley et al. showed how elderly travelers (those older than 50 years) were almost 10 times more likely to die from malaria compared to a younger cohort of tourists returning to the United Kingdom [91]. In addition, a study describing a cohort of travelers over the age of 60 years returning to Denmark with malaria had longer duration of hospitalizations and 2-fold higher parasitemias compared to younger patients [92].

Knowing this, it’s important to target elderly travelers going to malarious areas about the important of personal protective measures and to seek care early if they develop fevers upon return from travel. Recommendations for personal protective measures for insect vector avoidance (application of N, N-diethyl-3-methylbenzamide (DEET) to skin and permethrin to clothing) are not influenced by the age of the traveler [93].

Chemoprophylactic options include doxycycline, mefloquine, chloroquine and atovaquone/proguanil and all are generally well tolerated in elderly travelers, but there are some nuances based on side effect profiles. In light of polypharmacy in elderly patients, atovaquone/proguanil is an ideal agent to use as it has very few drug-drug interactions (Table 2) and is well tolerated. The drug requires no dose adjustment for age, has no difference in pharmacokinetics compared to younger patients and although the bioavailability of cycloquanil appears higher, there have been no serious toxicity reported in an older population. [94]. However, malarone cannot be used in elderly travelers with severe renal impairment (i.e. creatinine clearance <30 ml/min]) [94]. Mefloquine cannot be used in the setting of neuropsychiatric disorders or cardiac conduction disease [95]. For these reasons, this medication is no longer commonly prescribed to patients of all ages in the US. Chloroquine can be used in the elderly populations. However, due to concerns of of retinopathy and macular degeneration associated with this drug, it should be avoided when there are ophthalmological concerns at baseline, and periodic eye exams should be performed in the setting of prolonged use [96]. Doxycyline has concerns of dietary and supplement restrictions, pill esophagitis, and photodermatitis making it more difficult to give to older travelers [97].

## Conculsions

This guideline reviews a broad range of considerations when preparing an elderly patient for international travel. Providers should appreciate that older travelers have unique comorbidities with regards to cardiovascular, pulmonary, thromboembolic diseases and polypharmacy. They should use the travel visit as an opportunity to address both travel and standard vaccines with the understanding that an aging immune system has less of an immune response to immunizations. Finally, advanced age has an impact on the incidence and management of travelers’ diarrhea, jet lag, altitude illness and malaria prevention.

Admittedly, the field of travel medicine, especially pertaining to subgroups of more vulnerable travelers such as the elderly, is sometimes lacking in rigorous published evidence. Significant gaps in research include a better understanding of defining the exact components of pre-travel screening and the practical implications for immunosenescence when administering travel vaccines to the elderly. Further gaps include the optimal approach to travelers’ diarrhea in light of concerns for both drug-drug interactions and the emergence of drug resistant organisms in addition to jet lag prevention.

Despite the recognition that randomized controlled trials are needed in this field, this guideline reviews fundamental interventions, to the best extent of what the literature supports, when preparing elderly patients to safely participate in international travel.
